# Clinical, electroretinographic and histomorphometric evaluation of the retina in sheep with natural scrapie

**DOI:** 10.1186/1746-6148-7-25

**Published:** 2011-06-06

**Authors:** Alain Regnier, Olivier Andreoletti, Olivier Albaric, Delphine Cayez Gruson, François Schelcher, Pierre-Louis Toutain

**Affiliations:** 1 UMR 181 Physiopathologie et Toxicologie Expérimentales, INRA, Ecole Nationale Vétérinaire, 23 chemin des Capelles, B.P. 87614, 31076 Toulouse Cedex 3, France; 2 UMR 1225 Interactions Hôtes-Agents Pathogènes, INRA, Ecole Nationale Vétérinaire, 23 chemin des Capelles, B.P. 87614, 31076 Toulouse Cedex 3, France

**Keywords:** electroretinography, prion, retina, scrapie, sheep

## Abstract

**Background:**

The retina is part of the diencephalon in a peripheral location and may be involved in prion diseases. Retinal function and structural changes were assessed in naturally scrapie-affected red face Manech ewes presenting the classical signs of the disease, and clinically healthy age-matched subjects for controls. Ophthalmic examination was done prior to electroretinography (ERG), which was carried out under conditions that allowed photopic and scotopic activities to be assessed. Histomorphometry of the inner and outer retinal layers was performed *post-mortem*, and retinas were also examined for evidence of abnormal prion protein (PrP^Sc^) accumulation and glial fibrillary acidic protein (GFAP) upregulation as a marker of gliosis. Scrapie status was determined by examination of brain tissue

**Results:**

Ocular reflexes and ophthalmoscopy did not reveal any difference between scrapie affected and control animals. Although the light-and dark-adapted ERG responses of both rod-and cone-mediated functions had a similar waveform in scrapie-affected and control sheep, a significant reduction in the amplitude of the ERG a-and b-waves was observed in affected animals compared to controls. These functional alterations were correlated with a substantial loss of cells in the outer nuclear layer (ONL), lengthening and disorganization in photoreceptor segments, and substantial reduction in cellularity and thickness of the inner nuclear layer (INL). The degenerative changes in the INL and ONL were most marked in the central and paracentral areas of the scrapie retinas, and were accompanied in all scrapie retinas by PrP^Sc ^deposition in the ganglion cell and synaptic layers. GFAP immunoreactivity was mainly increased in the ganglion cell and inner plexiform layers.

**Conclusions:**

No appreciable fundoscopic changes were observed in the scrapie-affected ewes although reproducible changes in retinal function as measured by ERG were observed in these animals. The alterations in the receptoral and post-receptoral pathways corresponded to the degenerative lesions observed in the ONL and INL of the scrapie retinas. The retinal degeneration was associated with prion protein infectivity which presumably spread via the optic nerve.

## Background

Transmissible spongiform encephalopathies (TSE), or prion diseases, are fatal neurodegenerative diseases with a very long incubation period which include kuru and Creutzfeld-Jacob disease (CJD) in humans, bovine spongiform encephalopathy (BSE), scrapie in sheep and goats and transmissible mink encephalopathy [1,2]. Accumulation of an abnormal isoform (PrP^Sc^) of a normal cellular protein (PrP) in affected host tissues is considered a disease hallmark, and its deposition in tissues correlates with infectivity [3,4]. According to the prion hypothesis, PrP^Sc ^itself is thought to be the causative agent of TSE [5].

The retina is a part of the diencephalon in a peripheral location [6], and its involvement in the TSE context was initially explored in rodent models of CJD [7] and scrapie [8-11] before being documented in humans affected with the sporadic and variant CJD [12-14]. Previous studies assessing the retinal changes in sheep with natural scrapie have been performed, but without morphometric analysis [15,16], and information on the activity of the retina in scrapie-infected sheep is presently limited to one case report [17].

As a follow-up to our initial report [18], this paper further defines the functional and structural abnormalities of the retina in sheep with natural scrapie using ophthalmic, electroretinographic, morphometric, histopathological and immunohistochemical examinations.

## Methods

### Animals

Seventeen scrapie-affected red face Manech ewes at various stages of disease progression were collected from different field scrapie-infected flocks. They were between 1 and 3 years old. Clinical diagnosis relied on observation of classical scrapie signs (i.e. pruritus, behavioral changes, tremor, and locomotor incoordination). Six clinically healthy age-matched red face Manech ewes were used as controls. All animals were eventually subjected to euthanasia and the definitive scrapie status was determined by examination of brain tissue. All animal experiments have been performed in compliance with our institutional and national guidelines in accordance with the European Community Council directive 86/609/EEC. The experimental protocol was approved by the INRA Toulouse/ENVT ethics committee.

### Physical and electrophysiological examinations

An ocular examination including visual testing by the menace response and pupillary light reflexes, as well as indirect and direct ophthalmoscopy after pupil dilation with topical 0.5% tropicamide was performed. For the full-field electroretinogram (ERG) recordings, the ewes were placed in metabolism cages, and kept with a background room illumination of 27 cd.m^-2 ^(photometer S371R Optical Power Meter, Graseby Optronics, Orlando, FL, USA) for 2 hours. The animals were then anesthetized by intramuscular injection of ketamine (11 mg/kg) and xylazine (0.22 mg/kg). They were positioned in sternal recumbency with the head immobilized in a headrest by means of padded supports and straps. The muzzle was held horizontally, and the upper eyelid of both eyes was drawn back by placing 2 interrupted vertical mattress sutures. After topical anesthesia with 0.5% oxybuprocaine, a stainless steel recording needle was positioned subconjunctivally, 2-3 mm posterior to the limbus, at the 12 o'clock position. The reference electrode was placed subcutaneously at the base of the ear and the animal was grounded by another electrode placed subcutaneously in the occipital region. The cornea was kept moist by periodic topical administrations of a 0.1% hyaluronate sodium solution. The ERG responses were elicited simultaneously from both eyes, with stimuli of 200-μs duration generated by white strobe flashes. The flash units (Varéclat^®^, Alvar Electronic, Montreuil, France) were positioned 5 cm from each eye on the visual axis. The signals were fed back to an ERG recording system (MP3, ECEM électronique et informatique médicale, Ozoir-la-Ferrière, France), using analog bandpass filtering from 1 to 300 Hz in conjunction with 60-Hz notch filtering. For each recording, 8 consecutive responses were averaged to stimuli presented at 2 Hz. After the 2 hours of light adaptation, an initial cone-dominant response was elicited with unattenuated white light stimuli of 1.7 cd.s^-1^.m^-2^. The animals were then placed in the dark, and rod-dominant responses were obtained 1, 5, 10 and 15 minutes after adaptation to complete darkness, using a blue filter (Kodak Wratten 47B) and a neutral density filter (1.0 ND) in front of each flash. After 18 minutes of dark adaptation, a flash ERG response including both rod and cone components was recorded to unattenuated white flashes. Each recording session took about 20 minutes and during this period no additional anesthesia was applied. The amplitude and implicit time of the initial negative (a-wave) and/or the subsequent positive (b-wave) peak of the response were measured in the normal way. The amplitude of the a-wave represented the voltage difference between the baseline level and the peak of the first negative deflection, whereas the amplitude of the b-wave represented the voltage difference between the peak of the wave and the preceding trough [19]. Implicit times of the a-and b-waves were measured from the time of the light stimulus presentation to the peak of each wave [19].

### Histomorphometry

Within 1 to 7 days after ERG recording, animals were killed with intravenous injection of sodium pentobarbital followed by exsanguination. Eyes from the 6 healthy and 13 scrapie-affected subjects were prepared for light microscopic and immunohistochemical examination. In these animals, the eyes were enucleated rapidly postmortem, immersed in Zenker's solution for 2 hours, then washed in tap water for 2 hours and placed in 80°alcohol solution. After bisecting each eye along the vertical meridian, the two halves of the globe were dehydrated and paraffin embedded. Sections 2 μm thick including the optic nerve were made, dried overnight at 56°C, deparaffinized and rehydrated before being stained with haematoxylin-eosin. They were blind coded, and morphometric investigation of the retina was made by two observers (OA and OA) at three locations, as previously reported [8]. They included the central retina, immediately adjacent to the dorsal edge of the optic disc, the paracentral retina halfway between the optic disc and *ora ciliaris retinae*, and the peripheral retina immediately adjacent to the *ora ciliaris retinae*. At each location, the thickness of the photoreceptor segments (combined outer and inner segment length), outer nuclear layer (ONL), outer plexiform layer (OPL), inner nuclear layer (INL) and inner plexiform layer (IPL) was measured at x400 magnification, using a digital image analyzer (Visiolab 1000, Meylan, France). The thickness of each individual retinal layer was determined in pixels using an internal standard with an arbitrarily fixed pixel graduation. The relative number of cells present in each retina was also estimated at x400 magnification by counting the nuclei in ONL and INL respectively. For each predetermined site of the retina (central, paracentral and peripheral), ONL and INL cell densities from each eye were counted within three fields of 250 μm wide and these data were computed to a mean value.

### Immunohistochemistry

The tissue sections including the optic nerve area submittted to immunohistochemistry, were first denaturated with 98 per cent formic acid for 30 minutes at room temperature and then autoclaved for 20 minutes at 121°C. After endogenous peroxidase was quenched with H_2_O_2 _in methanol, the slides were blocked with 20% normal goat serum in TBS for 20 min and incubated with the primary antibody for 60 min at room temperature, followed by 30-min incubations first with biotinylated goat anti-rabbit immunoglobulins or goat anti-mouse immunoglobulins secondary antibodies diluted 1:100 and then with the streptavidin-peroxidase complex diluted 1:100. The reaction was developed using 3,3'-diaminobenzidine (ChemMateTM Detection Kit Peroxidase/DAB, K 5001, DAKO, Copenhagen, Denmark). Each step was followed by three 5-min washes using a 1% skimmed milk-0.05% Tween 20 in TBS. Finally, tissue sections were counterstained with Mayer's haematoxylin. PrP^Sc ^immunolabelling was carried out using a primary monoclonal antibody 2G11 raised in mice against recombinant peptide fragment R154-R171 of ovine PrP protein (generously provided by INRA VIM, Jouy-en-Josas, France) and diluted 1:1000 in ascite. The expression of the glial fibrillary acidic protein (GFAP), as a reactive marker of Müller cells gliosis, was determined using a primary polyclonal antibody, raised in rabbits against bovine GFAP (GFAP 0761, Dako, Copenhagen, Denmark).

### Confirmation of disease status

For each animal, the brain was removed, stored in 10% formalin, and processed for light microscopic and immunohistochemical examination of the obex and midpons as previously described [20].

### Statistical analyses

Nested analysis of variance with unequal sample sizes examined pooled data from both eyes for the significance of ERG response differences between diseased and control animals [21]. For the morphometric evaluation of the retina, three measurements of each variable were made in every location and averaged for statistical analysis. As contralateral eyes were not considered to be independent of each other, the statistical analyses were performed using information from one eye randomly chosen for each ewe. For each variable, the comparison between the diseased and control groups was made by using a one-way analysis of variance. For each analysis, the residuals from the fitted model were graphically assessed; in each case the standard assumptions of normality, constant variance and randomness were confirmed. Statistical significance was defined as p value < 0.05.

## Results

In the 17 clinically suspect ewes the diagnosis of scrapie was confirmed by observation of vacuolar changes and PrP^Sc ^accumulation in the brainstem. In contrast, brain tissues from the clinically healthy sheep showed no spongiform vacuolation, and no PrP^Sc ^deposits were detected in their lymphoid tissue and central nervous system.

During ocular examination, no mydriasis was detected in the scrapie-affected sheep and normal pupillary light reflexes were observed in both groups of animals. The palpebral and corneal reflexes were normal in all affected sheep, but inconsistent menace responses were observed in 1 control and 3 diseased sheep. No ophthalmoscopic evidence of fundic lesions was apparent in either the 17 affected ewes or the control animals.

The light-adapted ERG responses were recorded from all animals but the mean amplitudes of the cone-dominant a-and b-waves were significantly lower (p < 0.01) in scrapie affected ewes compared with controls (Table 1). Implicit time of light-adapted a-and b-wave responses was not significantly different between control and affected ewes (a-wave: 13.1 ± 0.7 ms *vs *13.7 ± 2.1 ms, p > 0.05; b-wave: 29.6 ± 0.9 ms *vs *28.4 ± 2.1 ms, p > 0.05). Following dark adaptation and using blue light stimulation, the a-wave of the rod-dominant ERG responses was not recordable in both the control and affected animals. With the adapting time duration the mean rod-dominant b-wave amplitude increased gradually in both groups of animals but remained significantly lower (P < 0.01) in affected than in healthy subjects for each time point (Table 1). The latencies of the cone-mediated b-waves were not statistically different between the control and affected animals at each time point. Mixed rod-cone responses to dark-adapted unattenuated white stimuli were recorded from all diseased ewes. The mean amplitude of the a-wave was significantly reduced (p < 0.05) in these animals compared with controls (Table 1). However, the difference in the b-wave amplitude between the two groups was not significantly different (p > 0.05) because of the large variability of the results in affected ewes (Table 1). The dark-adapted white flash stimuli induced no significant difference (p > 0.05) in implicit time between control and affected sheep.

**Table 1:** Results of the electroretinographic recordings

Stimulus and adaptation	Time (min)	a-wave amplitude (μV)		b-wave amplitude (μV)	
		
		Control	Scrapie	Control	Scrapie
wL	0	87.9 ± 10.2	70.6 ± 13.9**	342.1 ± 62.3	224.8 ± 86.9**
blD_1_	1			112.7 ± 25.8	48.9 ± 28.0**
blD_2_	5			271.1 ± 36.6	118.7 ± 49.9**
blD_3_	10			310.5 ± 31.1	169.6 ± 55.8**
blD_4_	15			343.2 ± 35.2	189.9 ± 59.1**
wD_5_	18	108.5 ± 19.2	88.1 ± 18.4*	257 ± 43.0	238.2 ± 100.7

Electroretinographic (ERG) response amplitudes (mean ± SD) obtained under various stimulus conditions in unaffected control and naturally scrapie-affected sheep. wL: white light stimuli on light-adapted eyes; blD: blue light stimuli on dark-adapted eyes; wD: white light stimuli on dark-adapted eyes. Level of significance *P < 0.05, **P < 0.01.

**Figure 1:**
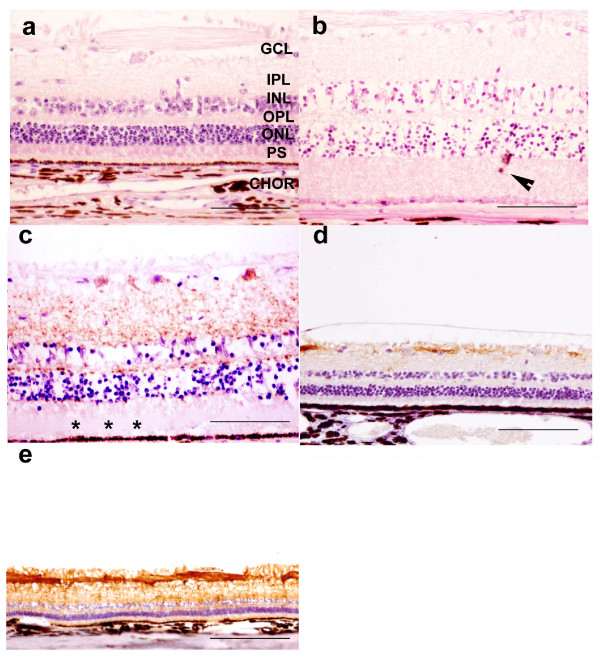
**Photomicrographs of retinal cross-sections of unaffected (a, d) and naturally scrapie-affected (b, c, e) sheep**. All layers of the control retina show normal organization and cytoarchitecture (a). Ganglion cell layer (GCL), inner plexiform layer (IPL), inner nuclear layer (INL), outer plexiform layer (OPL), outer nuclear layer (ONL), photoreceptor segments (PS) and choroid (CHOR). Bar: 100 μm. In the scrapie-affected retina, not only was there a disorganization and thickening of the PS layer, but also an overall decrease of cellular density within the INL and ONL (b). Arrowhead indicates displaced photoreceptor nuclei in the PS layer. Bar: 100 μm. Immnohistochemical detection of abnormal PrP deposits (peroxydase-DAB, brown deposits-2G11 monoclonal mouse antibody) in a scrapie retina (c). Asterisks indicate the amorphous material accumulated in the photoreceptor outer segment layer. Bar: 100 μm. Müller cells labelling (GFAP rabbit polyclonal antibody peroxydase-DAB, brown coloration) in the retina from a control (d) and a naturally-scrapie affected sheep (e). Bars: 300 μm.

Light microscopic examination of the control eyes revealed no retinal abnormalities (Figure 1a). In all scrapie affected sheep, histological lesions of the retina were observed but with variable severity among subjects. In the outermost retina, the elongation and disorganization of the photoreceptor segments in the scrapie retinas (Figure 1b) resulted in a significant thickening of this layer (p < 0.05) compared to the controls (Table 2). A multifocal accumulation of amorphous eosinophilic material between the photoreceptor outer segments and the retinal pigment epithelium, which corresponds to the subretinal space, was also observed in the affected animals (Figure 1c). The retinal pigment epithelium appeared histologically normal. In contrast, a widespread decrease in thickness of the ONL was found in the retina of affected sheep, along with a reduction in the number of photoreceptor nuclei. These changes were more marked in the central and paracentral regions than in the peripheral retina (Tables 2). Large perinuclear spaces suggested loss of cytoplasm and cell shrinkage. Presence of occasional pyknotic nuclei in the ONL provided further evidence of photoreceptor cell degeneration. Displacement of photoreceptor nuclei in the photoreceptor inner segment layer was sometimes observed (Figure 1b). In retinas from scrapie-affected animals, the changes in the OPL manifested as an irregular thickness with a significant thinning in the central and paracentral areas of the retina (Table 2). This was associated with a clear disorganisation of OPL in about half of the specimens. A significant thinning in the INL was observed in the retina of the scrapie-affected animals (Table 2) in association with a significant decrease in nuclei density (Table 3), and a moderate cellular infiltration by macrophage-like cells. There was no statistically significant difference between the IPL of scrapie and control retinas (Table 2), but scattered areas of vacuolation were observed in the scrapie retinas displaying the most severe morphological changes. Because the ganglion cell density varied considerably throughout the control retinas, comparative morphometric investigation could not be done. However, there was no apparent morphologic difference in this retinal layer between the two animal groups.

**Table 2:** Average thickness of retinal layers

Layer	Central retina		Paracentral retina		Peripheral retina	
	
	Control	Scrapie	Control	Scrapie	Control	Scrapie
Photoreceptor segments	13.5 ± 1.1	17.0 ± 4.3***	13.8 ± 0.7	18.3 ± 3.6***	14.6 ± 1.9	18.2 ± 3.1***
Outer nuclear	28.6 ± 1.7	24.0 ± 6.0*	27.2 ± 0.9	24.4 ± 3.6*	24.4 ± 1.6	24.3 ± 3.9
Outer plexiform	7.2 ± 0.5	5.9 ± 1.7*	7.8 ± 0.7	7.5 ± 2.7**	8.4 ± 1.2	7.9 ± 3.0*
Inner nuclear	20.7 ± 1.5	17.1 ± 3.1**	19.7 ± 1.3	17.1 ± 2.8*	19.9 ± 1.6	17.6 ± 2.9***
Inner plexiform	30.0 ± 3.1	32.9 ± 6.5	33.5 ± 2.0	32.7 ± 4.6	32.5 ± 2.3	31.9 ± 5.6

Thickness of the retinal layers (in pixels) in unaffected control and naturally scrapie-affected sheep. Data are expressed as mean ± SD. Level of significance *P < 0.05, **P < 0.01, ***P < 0.001.

**Table 3:** Cell density measurement in ONL and INL

Nuclei/field	Central retina		Paracentral retina		Peripheral retina	
	
	Control	Scrapie	Control	Scrapie	Control	Scrapie
Outer nuclear layer	5.9 ± 0.7	4.7 ± 1.1**	5.8 ± 0.6	4.9 ± 0.8*	5.3 ± 0.9	5.1 ± 1.5
Inner nuclear layer	1.5 ± 0.2	1.0 ± 0.2**	1.4 ± 0.2	1.0 ± 0.2*	1.5 ± 0.2	1.3 ± 0.4

Cell density measurement (mean ± SD) in outer nuclear layer (ONL) and inner nuclear layer (INL) of retinas from unaffected control and naturally scrapie-affected sheep. In each of the three areas examined from each retina, the cell count was obtained by averaging the measurements made within three fields of 250 μm wide and at x400 magnification. Level of significance *P < 0.05, **P < 0.01

No PrP^Sc ^immunolabelling was observed in the retinas of the control sheep but, conversely, PrP^Sc ^deposits were observed in the neuroretina of all the scrapie-affected cases and were more prominent in the central area of the retina. However, in animals with the strongest positive reaction, the deposits were more homogenously distributed throughout the retina. PrP^Sc ^deposits were observed in the ganglion cell layer, IPL and OPL of each affected retina, and in the inner segments of the photoreceptor layer of some of them (Figure 1c). The immunostaining took the form of a brownish granular pattern in the plexiform layers of the neuroretina and perikarya of the ganglion cells (Figure 1c). Positive PrP^Sc ^immunostaining was not evident within the optic nerve or any ocular tissues other than the neuroretina. Compared to the control retinas (Figure 1d), an increased expression of GFAP was observed in the IPL and ganglion cell layer from all scrapie retinas, indicating Müller cell gliosis (Figures 1e). In the cases where PrP^Sc ^accumulation was the most intense, increased GFAP immunoreactivity was also present in the INL and ONL.

## Discussion

Our results demonstrate that electrophysiological and histopathological changes indicative of retinal degeneration were present in all the scrapie-affected sheep examined for the current investigation. Nevertherless, no behavioral or neuro-ophthalmologic changes were found that were indicative of visual impairment in these animals. This is in agreement with recent reports in which deterioration of vision is never mentioned as a presenting sign of typical and atypical cases of natural ovine scrapie [20,22-25]. Differentially, blindness appears to be a major clinical feature in other ovine neurodegenerative diseases such as ceroid-lipofuscinosis [26] or *Helychrysum argyrosphaerum *poisoning [27]. The ocular fundus examination was unremarkable in our affected animals and did not reveal the rare and typical raised, roughly oval, blister-like areas previously described in two cases, which presumably result from accumulation in the subretinal space of the amorphous material noted morphologically [15]. Such ophthalmoscopic characteristics have never been described in other experimental or spontaneous TSE cases [7-9,11,13,14,28] and were not identified in our affected animals, possibly because the accumulation of the amorphous material, as seen histologically, was less pronounced in these animals than in the two cases reported by Barnett & Palmer [15].

Since the ovine retina is of mixed rod and cone type [29], the ERG responses obtained in our sheep were characterized by an obvious a-wave and a fast b-wave in light-adapted condition, and by a non recordable a-wave and a slow b-wave which increased in amplitude during dark adaptation [19]. In the control eyes, these responses were in agreement with those previously reported in healthy sheep studied under different protocols [30-32]. In the scrapie-affected animals, the significant reduction in the a-wave amplitude observed under photopic and scotopic conditions (about 20% compared to the controls) was indicative of abnormalities in the photoreceptor layer involving both the cone and rod mechanisms [33]. The degree of ERG a-wave amplitude reduction agreed well with the amount of morphologically determined photoreceptor nuclei loss in the ONL. The rod system dysfunction was especially demonstrated in recordings of the responses to blue light during the 15 minutes of dark adaptation, which showed a 45-56% reduction in the response amplitude when compared with controls. The reduction in amplitude of the b-wave, which represents contribution of the bipolar and Müller cells [34,35], was consistent with the changes of the bipolar cell layer noted in the retinal histopathology of the affected animals. Similarly, the ERG abnormalities detected in humans with CJD are characterized by a more marked decrease in the b-wave than a-wave amplitude [12,36]. Despite the reduction in amplitude, both rod-and cone-derived responses retained basically normal waveform and implicit times. This result is somewhat surprising in light of the morphological lesions, but a similar situation has been observed in mice with experimental scrapie [11]. It indicates that both the photoreceptors and the post-receptor pathways retain a modicum of normal function [19,37-39] and is in agreement with the uneven distribution of the histological lesions in the retinas of our affected sheep, which probably left a sufficient number of functional retinal neurons.

Morphological changes representative of retinal degeneration and consistent with the ERG findings were observed in all scrapie-affected sheep in the current study. These histopathological lesions were associated with PrP^Sc ^deposition in all of the cases and alterations in the retinal glia manifested as an increased expression of the intermediate filament GFAP. In a recent report, only 50% of sheep with signs of natural scrapie were found to have retinal degenerative lesions [16]. In our affected sheep, the multifocal accumulation of amorphous material within the subretinal space was less marked than in the two cases reported by Barnett and Palmer [15]. This material, composed of a complex lipid which is probably disintegrating outer segments [15], was associated with elongation and disorganization of the photoreceptor segments. These alterations were clearly a consequence of the photoreceptor degeneration that was also reflected by the reduction in thickness and cellularity of the ONL. A complete loss of photoreceptors similar to that described in the retinas of scrapie-infected mice [9,11], and hamsters [8,28] was never observed, but as with the rodent scrapie models, the ONL changes in the affected sheep were most severe in the central area and were occasionally associated with extrusion of photoreceptor nuclei to outer limiting membrane. The changes observed in the inner retinal layers of our affected sheep were characterized by a significant reduction in the cellular density of the central and paracentral INL, a slight influx of phagocytic cells (presumably macrophages) in the INL, and a few vacuolar changes within the IPL. Reduction in INL cellularity has been found in hamsters with experimental scrapie infection [8], and vacuolization of the plexiform and ganglion cell layers is a striking feature of the retinopathy associated with CJD in humans [12]. Although retinal degeneration is a recurrent pathological feature of scrapie, the morphological changes identified in the neurosensory retina of our affected sheep showed differences with previous observations made both in the rodent models [8,9,11,28], and natural cases in sheep [15,16]. These discrepancies may reflect the species and breed of the host and/or the biological properties of the scrapie infecting strain, as well as the time course of the disease, as previously underlined [7,40].

Although the process of the retinopathy associated with the TSE has not been fully established, some mechanisms and hypotheses have been put forth. The current hypothesis, based on the study of the retina in sheep and hamsters with scrapie [8,10,16,28,41] as well as in humans with sporadic and variant CJD [13,14], combined with the observation that the scrapie agent can be transported along a direct pathway from the superior colliculus to the retina [42], is that PrP^Sc ^can spread from the brain to the retina via the optic nerve. Although immunoreactivity was not detected in the optic nerve of the current affected sheep, our observation that the histological changes were the most severe in the central and paracentral areas of the retina is in agreement with the hypothesis of neural spread. The possibility that scrapie infection spreads from the central nervous system to the retina by other modes of propagation, such as via the extracellular space or within and between glia, has not been ruled out [42]. Irrespective of the way the infection travels to the retina there is evidence that scrapie prions are present and replicate in the neurosensory retina prior to development of retinal lesions [16,41,43]. In the current study, PrP^Sc ^accumulation was identified in all the scrapie retinas, and its localization within the retinal layers was in agreement with previous observations in sheep naturally [16] and experimentally [44] infected with scrapie. The presence of the PrP^Sc ^in the plexiform layers, which are the high synaptic density areas of the retina, as seen in sheep with scrapie [16] and humans with CDJ [13], also argues for a centrifugal spread from the brain to the eye via the optic nerve [14]. The loss of cells contributing to the outer and inner nuclear layers of the retina in subjects with TSE presumably occurs by apoptosis in response to exposure to PrP^Sc ^and/or its degradation products as found in the rat retina with the PrP^Sc ^fragment P106-126 [45]. Recent findings in sheep experimentally infected with scrapie demonstrate a decrease in immunoreactivity of specific markers of bipolar, ganglion and Müller cells [43]. These neuronal functions were impaired before the onset of microscopically retinal pathology was detected, suggesting that alterations in protein expression patterns in retinal cells might be one of the pathways for inducing retinal degeneration [43]. Gliosis is a fundamental response of the central nervous system to scrapie infection in sheep [40], and the current and previous findings [16,43,45] demonstrate that it is a prominent pathological feature of the retinopathy in affected animals. The gliotic response of Müller cells is known to occur in various retinal diseases and may increase the susceptibility of neurons to stressful stimuli in the diseased retina [46]. In the case of ovine scrapie, whether the retinal gliosis participates in the death of neurons in the retina or is a secondary event resulting from the loss of the retinal neurons remains to be elucidated.

## Conclusion

An extensive retinal degeneration was observed in all these ewes with naturally occurring scrapie although no clear visual impairment and no appreciable fundic alterations were observed in these animals. The characteristics of their ERGs indicated that both the rod and cones responses were significantly reduced in agreement with the retinal lesions which predominated in the outermost layers. Scrapie prion accumulation was present in all scrapie retinas, and both the marked loss of photoreceptors and bipolar cells and the strong PrP^Sc ^staining within the central retina were consistent with a centrifugal spread of scrapie infectivity. One of the main problems with the TSE therapeutic trial is the quantitative characterization of the progression/regression of neurodegenerative process, in treated versus untreated individuals. In most of the cases, evaluation of therapeutic efficacy relies on the quantification of different lesions (neuronal loss/abnormal PrP accumulation) in tissues, which requires killing of the subjects. Because it is a non-invasive procedure that directly reflects neuroretinal degeneration, electrophysiological follow-up of the retinal function in TSE affected animal will warrant further studies to know if it may provide a reliable insight for monitoring evolution/regression of neurodegenerative changes in individuals.

## List of abbreviations

BSE: Bovine spongiform encephalopathy; CJD: Creutzfeld-Jacob disease; ERG: Electroretinography; GFAP: Glial fibrillary acidic protein; INL: Inner nuclear layer; IPL: Inner plexiform layer; ONL: Outer nuclear layer; OPL: Outer plexiform layer; PrP: Normal prion protein; PrP^Sc^: Abnormal prion protein; TSE: Transmissible spongiform encephalopathies

## Authors' contributions

AR developed study design, contributed to ophthalmologic and electroretinographic investigations, performed analysis of ERG data, and took part in writing the manuscript. OAn performed histomorphometric and immunohistological examinations, and took part in writing the manuscript. OAl was involved in histomorphometric and immunohistological examinations. DC was involved in ophthalmologic and electroretinographic investigations. FS contributed to the study design and critically revised the manuscript. PLT contributed to the statistical analysis of the data and took part in writing the manuscript. All authors read and approved the final manuscript.
